# Developing lay health worker policy in South Africa: a qualitative study

**DOI:** 10.1186/1478-4505-10-8

**Published:** 2012-03-12

**Authors:** Karen Daniels, Marina Clarke, Karin C Ringsberg

**Affiliations:** 1 Health Systems Research Unit, Medical Research Council, PO Box 19070, Tygerberg 7505, South Africa; 2 Nordic School of Public Health, NHV, Box 12133, SE 402 42 Gothenburg, Sweden; 3 Nursing Division, Stellenbosch University, Francie Van Zijl Drive, Tygerberg, 7505, South Africa

**Keywords:** Lay health workers, Health policy analysis, Gender, Qualitative research

## Abstract

**Background:**

Over the past half decade South Africa has been developing, implementing and redeveloping its Lay Health Worker (LHW) policies. Research during this period has highlighted challenges with LHW programme implementation. These challenges have included an increased burden of care for female LHWs. The aim of this study was to explore contemporary LHW policy development processes and the extent to which issues of gender are taken up within this process.

**Methods:**

The study adopted a qualitative approach to exploring policy development from the perspective of policy actors. Eleven policy actors (policy makers and policy commentators) were interviewed individually. Data from the interviews were analysed thematically.

**Results:**

Considerations of LHW working conditions drove policy redevelopment. From the interviews it seems that gender as an issue never reached the policy making agenda. Although there was strong recognition that the working conditions of LHWs needed to be improved, poor working conditions were not necessarily seen as a gender concern. Our data suggests that in the process of defining the problem which the redeveloped policy had to address, gender was not included. There was no group or body who brought the issue of gender to the attention of policy developers. As such the issue of gender never entered the policy debates. These debates focused on whether it was appropriate to have LHWs, what LHW programme model should be adopted and whether or not LHWs should be incorporated into the formal health system.

**Conclusion:**

LHW policy redevelopment focused on resolving issues of LHW working conditions through an active process involving many actors and strong debates. Within this process the issue of gender had no champion and never reached the LHW policy agenda. Future research may consider how to incorporate the voices of ordinary women into the policy making process.

## Introduction

### LHWs in the international and South African health care context

Lay Health Workers (LHWs) have been defined as any health worker carrying out functions related to health care delivery; trained in some way in the context of the intervention; and having no formal professional or paraprofessional certificated or degreed tertiary education [1]. Internationally LHWs have come both in and out of favour over the past 50 years [2]. They can be found in almost every primary health care system across the world, carrying out a range of tasks including palliative care, counselling, health promotion, treatment support, breastfeeding support etc [2].

The current re-emergence of LHW programmes in low- and middle- income countries (LMICs) over the past decade occurs within a context in which many health systems are burdened by severe health worker shortages and the inequitable distribution of health staff [3-5]. The World Health Organisation (WHO) has estimated that 57 countries face critical health worker shortages [6]. Thirty six (63%) of these countries occur in Sub-Saharan Africa [6]. This crisis has been exacerbated by the stress which the demand for HIV care puts on already overstretched health systems [3,7,8].

The crisis faced by other LMICs has not escaped South Africa. On the face of it South Africa is not short of key health care staff with a density of 4.9 physicians, nurses and midwives to 1,000 population [9] (WHO critical threshold is 2.3 = 1,000 [6]). However, these human resources are distributed in favour of privately paid for care and urban institutions, threatening the delivery of key health programmes [4]. Although there is a long history of LHW programmes in South Africa [10] (see Table 1), health worker shortages [11] and the demands of HIV care [10,12] have increased demand for such programmes in recent years. It is estimated that between 38 500 and 65 000 LHWs, who are mostly women, operate either as unpaid volunteers, stipended volunteers or fully employed workers within the country [11]. The expansion of this workforce prompted the need for government intervention and thus since 2003 the South African government has been developing and implementing national policies for LHW programmes [13]. Within the current health system LHWs are not employed directly by the state [11,12]. Instead the national government makes funding available to provincial governments who in turn fund LHW programmes run by Non Governmental Organisations (NGOs) and Not for Profit Organisations (NPOs) [11,14]. There are however variations in how individual NPOs and NGOs are funded, to whom they are accountable and the roles of external donors [14].

**Table 1:** Brief Timeline of LHW projects and historical landmarks in South Africa

Date	Event
1913	Natives Land Act (7.3% of South African land dedicated for Africans' habitation) *Signals a start of repressive racial legislation*

1930s	Lay people trained as malaria assistants

1948	Nationalist Party comes to power

1940's-1990's	LHW projects emerge as a response to an healthcare system which was intentionally inequitably distributed under an apartheid regime *Notably LHW projects remain outside of government health system*

1990	ANC/PAC unbanned *Signals the start of the end of apartheid*

1992-1994	Preliminary ANC health plan; national LHW workshops/conferences

1994	First democratic elections (Mandela elected) *Government adopts district health system which does not include LHWs*

post 1994	Many former LHW projects collapsed

Late 1990s-2000's	Uncoordinated re-emergence of LHW projects mainly within healthcare for people living with HIV/AIDS

2003/4	National Community Health Workers Policy Framework *First formal recognition of LHWs by post apartheid government*

2004	The Expanded Public Works Programme Social Sector Plan 2004/5 - 2008/9 (includes LHWs as home based workers within Public Works Programme)

mid-late 2000's	Continued redevelopment of LHW policy

November 2009	Release of Community Care Worker Management Policy Framework(Draft Version 6.0, October 2009)

Adapted from van Ginneken et al, pp. 1110-1111, [10]

### Gender and LHW implementation

A number of reviews and studies have pointed to the complexity of implementing LHW programmes including pointing to challenges experienced by LHWs themselves [2,15-17]. One area of complexity is the gender dynamics within LHW programmes [18,19]. The work carried out by LHWs and in particular the home based care aspect of the work (i.e. care delivered to the patient in the context of their home by a non-family member) has been described as care work [20,21]. In South Africa such care work is mostly done by women and it is also women who are mostly in need of care [20]. In the division of labour, men are engaged in physical tasks with women assigned to nurturing tasks [22].

As care workers, women make up the majority of LHWs in South Africa [11,12,16,22,23]. It has been repeatedly pointed to that these women face difficult and ambiguous working conditions [12,20,23-26]. This includes uncertainty regarding whether LHWs should be regarded as volunteers or full members of the health system and questions around whom LHWs are answerable to, given that they are funded by the state but employed by NGOs [13,14,23]. Short-term funding from national government and/or donors also results in the impermanence of LHW employment [20]. The difficulties with their working conditions are further contributed to by poor regulation [23] and misunderstanding of LHW policy by programme implementers [13]. These conditions, it is argued, limits the rights of LHWs, opening them to exploitation and low pay, and making it difficult for labour unions to organise them [23]. In combination the nature of the caring task and the difficult working conditions challenge female LHWs in particular with physical, emotional, social and economic stress [16,22,24,27]. In turn, LHWs who are not adequately cared for may deliver a less than adequate service [28].

### Gender sensitive policy making

State policies can however be designed to be gender sensitive [29] and to protect caring and care workers [21]. At an international level the WHO supports gender sensitive policy making in health [30]. Locally, South Africa has a national Gender Policy framework [31,32] and the Department of Health has its own gender policy [33]. Within the Department of Health oversight of the implementation of this policy is assigned to the office of the Gender Focal Point or Gender Desk. The South African government also contributed to the 53 rd session of the Commission on the Status of Women and is a signatory to its conclusions on the equal sharing of responsibilities between women and men, including caregiving in the context of HIV/AIDS [34]. Thus in principle the State recognises the need for gender equity in HIV/AIDS care for which LHWs are largely responsible at a home and community level.

### Policies regulating LHWs in South Africa

This support for gender equity at a national and departmental policy level is not congruent with the recent experience of implementation of LHW programmes and policies. Some of the difficulties with LHW programmes, including those related to gender, have been observed during the course of the implementation of two LHW policies [13,23,26,28]: the National Community Health Workers Policy Framework, 2003 and The Expanded Public Works Programme Social Sector Plan 2004/5--2008/9. The implementation of these policies began in 2004. In November 2009 a third policy, the Community Care Worker Management Policy Framework (Draft Version 6.0, October 2009) was released for comment by the Human Resources Directorate who was tasked with its development. This paper focuses primarily on the processes around the design of this draft policy framework (2009) as this was contemporary during our study period. Although the policy was not yet finalised, exploring the processes around its development offered insight into the agenda setting phase of policy making [35]. The issues around the implementation of the first two policies are covered in the earlier literature [12,13,23,26,28].

### Aim

This paper aims to explore the contemporary development of LHW policy in South Africa and to explain if and how gender was considered in this process.

## Methods

### Design of the study

A qualitative design was adopted in order to understand the phenomena under investigation as experienced by the actors involved in it. Exploring actors' own accounts of their experiences enhances understanding of the processes, rather than merely the outcomes, of policy development.

#### Data collection

The first author (KD) conducted face to face in-depth interviews with eleven key policy actors. Two of the interviews were co-conducted with a second interviewer (Inger Scheel, a senior researcher) who facilitated access to these particular informants.

A first pilot interview was conducted in April 2008 and the other 10 were conducted between September 2009 and August 2010. Each informant was approached in advance of the interview via email and telephone communication. At the start of each interview the topic was again explained and their formal signed consent was requested. The interviews took place at the office or home workspace of each of the informants (as per their preference). On average the interviews lasted for about an hour (range 30-105 minutes). The interviews were conducted in English which was the professional language of all informants, but a first language for only half of them.

The interviews were digitally recorded, and later transcribed verbatim by a professional transcribing service. The transcripts were checked for accuracy by KD who read and corrected each one while listening to the voice recording. The verbatim transcript formed the basis for the data set and analysis of the interview texts.

An interview guide was used with open thematic questions structured around the specific informant (e.g. policy developers were asked about their policy development experience, while policy commentators were asked about the experience of trying to engage with government around policy). The interviews were sufficiently open so as to allow informants to speak freely and share their insights on LHWs, LHW policy development and the issue of gender in implementation, in policy and in policy development. This enabled us to get various conceptions of the phenomena but they all focused on the issue of LHWs, policy development and gender from the perspective of the informants' experience and insights. Examples of questions: Currently the Community Care Worker Management Policy Framework is at draft version 6.0, can you share with us the status of this document and clarify both the process which has led up to it and the process which will follow; in your opinion who are the key people in this process and how have they contributed to it.

#### Informants

The informants were purposively sampled meaning that they were selected on the basis of their involvement and knowledge of the policy making process [36]. The initial selection was based on our knowledge of who the policy actors are, but further actors were included as our knowledge of the context expanded. The authors strove to include as wide a range of perspectives as possible [37]. For example we sought to find informants who were in favour of the policy and those who were critical of it.

Since the regime change of 1994 in South Africa there has been a common understanding that policies created by government should have some input from civil society. Therefore policy actors in this study were defined more broadly than only including government officials. The eleven interviews comprised of informants employed both inside and outside of the Department of Health:

• Policy makers (Department of Health):

○ Executive health managers (national and former provincial) (2) (EM)

○ National Department of Health officials, including technical policy writers/developers (DoH) (3)

• Policy commentators (Outside Department of Health)

○ Researchers (public health, gender and policy) (5) (Res)

○ Nongovernmental organisation director (1) (NGOD)

Given the relatively small number of stakeholders involved in this policy issue, it is not possible to provide more informant detail without compromising anonymity.

#### Analysis

Data were analysed by Thematic Content Analysis (TCA) [38,39]. It was mainly on manifest level [39] aiming at identifying, describing and thematisizing the gathered data. The transcripts were read and reread to obtain a sense of the whole. Each interview was regarded as a unit of analysis. Meaning units were identified in each interview. Thereafter meaning units from all interviews with similar content were combined into four themes: Working conditions as a key policy redevelopment driver; Actor engagement in the policy redevelopment process; Ideas and debates contemporary to the policy redevelopment process; and Gender considerations in the policy redevelopment process. The findings are presented under these themes.

#### Trustworthiness

In this study the authors sought to ensure that the research process was trustworthy, authentic and dependable in order that the findings would be a credible reflection of reality [40]. Several measures were taken to establish trustworthiness [41]. These included the three authors continuously discussing the design of the study, the thematic question guide, the analysis and the findings. The first author had the main responsibility but the second and third authors read selected transcripts and all authors were actively involved in the writing process. Complementary research competencies and experiences among the three authors influenced data interpretation and strengthened the rigour of the study. Quotations are also included to provide the reader with the opportunity to interpret data and thus establish confirmability.

#### Ethics

The study received approval from the ethics committee at the Medical Research Council of South Africa (#EC07-007). All informants in the study signed a consent form after being given a written study information sheet and a verbal explanation of the consenting process. Personal identifiers have been removed or disguised so that the informants are not identifiable.

## Findings

### Working conditions as a key policy redevelopment driver

All informants noted that the key issue that the new policy had to address was resolving the workforce problems born out of the experience of implementing the 2003 & 2004 LHW policies. They described a range of implementation problems including the policy being misinterpreted by implementers in the field. These problems, informants suggested, resulted in working conditions for LHWs that were appalling, chaotic, corrupt, mismanaged, unstable and essentially exploitative:

The conditions in which they work are appalling most of the time that they very often have to fund for the needs of the sick people from their own pockets, like buying food or stuff that people may need. Where they work for government the payment maybe in arrears by two or three months and the payment is not very high (Res).

Those informants (DoH) responsible for redeveloping policy saw this process as a means of correcting the implementation problems so as to improve the working conditions of LHWs. Furthermore they stated that since the concern was with the working conditions of LHWs, the Human Resources (HR) Directorate within the Department of Health and specific individuals within the directorate were viewed as the appropriate persons to find a policy solution and to drive this policy redevelopment process.

This has come about mainly because of the HR working conditions of community caregivers on the ground; now the reason why HR is involved it's because they are more to do with HR issues... that's why it is not located here [referring to a health programme], it is located there under Strategic Health Programmes where HR and working conditions is their core competency (DoH).

In resolving these working conditions problems, informants (DoH) told that members of the HR Directorate preferred to get firsthand knowledge of the policy implementation process by visiting the field themselves and speaking with LHWs. Thus informants (DoH) explained that the HR directorate arranged meetings with LHWs in four of South Africa's nine provinces. One informant (DoH) described these field visits as part of a process of Action Research, suggesting that the policy content was informed by the policy developers own research.

### Actor engagement in the policy redevelopment process

From almost all informants it became clear that there was a general expectation amongst people interested in LHW policy that the policy redevelopment process should involve the engagement by government of all stakeholders or policy actors. All informants made it clear that there was a wide range of policy actors. These included the Department of Health and within that the HR Directorate, various programme managers and the office of the Gender Focal Point; the Department of Social Development; the Department of Labour; LHWs; and civil society. It was explained in the interviews (DoH, Res, EM, NGOD) that the HR Directorate within the Department of Health held the responsibility of engaging this wide range of actors through consultation:

Yes they've been having many workshops that [referring to the policy writer] has been coordinating and [referring to a public health researcher] has been part of that process (EM).

#### Government departments as actors

The informants (DoH) told that programme representatives from within the Department of Health and representatives of the Department of Social Development were directly involved in informing policy content. Indirectly, the Department of Labour also influenced policy content because informants (EM, DoH) suggested that the Department of Health felt under pressure to adhere to labour laws. However as the quote below suggests the Department of Health did not always regard this as feasible or affordable:

Department of Labour has specified that if you employing people regardless of *who's *employing them, if they are employed to do a piece of work for you for X number of hours the minimum conditions of service apply... uniforms in some instances, leave days, maternity leave... You see and that's where it becomes a big problem for us. When you add up all of these things you get a bill that you then can't afford (EM).

#### Politicians as omniscient but invisible actors

Although politicians were not directly engaged in redeveloping the policy content, informants (EM, DoH) suggested that politicians set the framework within which the Department of Health operated. Informants suggested that the politicians influenced how LHWs would be employed in the health care system they made the decisions around budgets and resource allocation. The quote below highlights the constraints set by politicians:

... we had two mandates that were very problematic for us; the one was [referring to LHWs] had to be outside the department, within NGOs and from the NGOs they would work in the services.... The second mandate was that they would not be employed by the department... It was a political mandate... so what we sought then was to reverse the political mandate because we realised it was not gonna help us because we were just clashing [referring to civil society]... the main thing was really to say we needed the mandate to be removed and how we knew the mandate was a political mandate, because when I asked to remove it, I was told it was a political mandate and that would have to shift (DoH).

#### Engagement with civil society

Informants (Res, DoH, EM, NGOD) spoke of three sets of actors from civil society engaged in this policy redevelopment process--NGOs/NPOs, researchers and labour unions. While informants (Res, DoH, EM, NGOD) suggested that researchers and labour unions engaged with policy redevelopment only as policy commentators, NGOs/NPOs were described as recipients of government funding, targets of the policy and as policy commentators. One informant (DoH) suggested that as recipients of Department of Health funding NGOs/NPOs had to comply with policy stipulations:

Well the stick is simply our money, 'you either do it our way or you don't get our money' (DoH).

Civil society was described as trying to influence policy content through comments, opinions expressed in meetings and letters to government (NGOD, DoH, EM, Res). However some informants (Res, NGOD) relayed unhappiness with the level of engagement that had taken place, describing the process as frustrating both for the policy developers and civil society. Some informants (Res) felt that civil society was not adequately consulted:

I think then the big problem has been that *they *[referring to a section of the Department of Health] develop the new policy very much in isolation and without *real *consultation. There *were *a couple of consultative events; I don't think that it was consultation. I think that was sort of information sharing 'that is what we want to do' (Res).

Others (DoH) expressed a perception that the civil society criticisms were preventing finalisation of the policy and described this as creating a "paralysis" in the process. Informants (DoH, NGOD, Res) also expressed concern that the breadth of civil society meant that larger organised NGOs had a stronger voice in the process than smaller unorganised or individual NGOs (NGOD, Res, DoH).

#### LHW involvement in the process

The extent to which LHWs themselves were engaged in the policy redevelopment process was unclear from the interviews. Several informants (Res) suggested that LHWs were unorganised and voiceless. One informant (DoH) suggested that in the comments to government LHWs were only represented through the views expressed by civil society on their behalf, rather than through their own endeavours. Therefore this informant (DoH) explained that four provincial meetings were organised so that the policy developers could engage with LHWs directly. This informant furthermore suggested that the views and experiences expressed in these meetings influenced how the policy writers developed the policy. However, the extent to which these meetings were representative of LHWs broadly was unclear from the interview.

### Ideas and debates contemporary to the policy redevelopment process

The interviews offered insight into the kinds of ideas that were being debated during the course of the policy redevelopment process. The key areas of debate were around who should be responsible for care, whether LHWs should be specialist or generalist, whether they should volunteer or not and whether they should be employed by government or not. These ideas may not have been directly translated into policy content but it is important to know what the contemporary thinking was.

#### Responsibility for care

One of the key debates emerging from the interviews was around whether LHWs and home based care programmes appropriately distributed responsibility for caring between government and households. One informant (Res) suggested that home based care shifted the responsibility of care onto households and the women within those households. This informant furthermore suggested that the cost of keeping the ill in hospital was higher for government than the cost of paying LHW stipends. In contrast, another informant (DoH) suggested that it was appropriate to support "ordinary people" in caring for the ill at home and that government should not be the sole provider of health care. This informant further expressed the idea that there should be a partnership between government and communities in service delivery. But the accountability and responsibility within this partnership was questioned by another informant:

On one hand we are saying there should be community ownership, very often there should be volunteers, they should be accountable to communities, at the same time we *expect *them to render *essential *health services increasingly, hmm, which in my *view *is a government function (Res).

#### What model would work best

Another key debate described by the informants (EM, Res, DoH) focused on which model would best serve both government needs and address LHW working conditions. Informants collectively spoke of two key issues, firstly whether or not LHWs should be specialist or generalist health workers and secondly whether or not they should be volunteers. One informant (EM) suggested that the government preferred generalist LHWs who dealt with a range of issues at a household level and that this preference is reflected in the draft policy content. Another informant (DoH) argued that research contemporary to the policy redevelopment presented evidence supporting specialists but that in the informant's (DoH) experience LHWs in the field had to work as generalist. When discussing the different models, one informant said:

We simply don't know enough about it (Res).

Informants (EM, DoH, Res) made it clear that the model of LHWs as unpaid volunteers was no longer regarded as appropriate. However there was disagreement in informants' accounts as to whether the revised policy went far enough in changing this (Res, DoH). The final decision was however regarded by one informant as being a political one:

Civil society wants it to be a professional cadre you know? Whereas I think the officials want it to be more volunteer.... and ultimately the politicians will decide what they want (EM).

#### Who should employ LHWs

By far the largest issue being debated as described by the informants (EM, DoH, Res, NGOD) was in relation to what was referred to as the 'externalisation' of community health work. Externalisation was explained by informants (Res, NGOD) as the policy position that government would support LHWs through funding their salaries or stipends but would not absorb them into the health system as formal government employees. Some informants (EM, Res) suggested that government took this position because it was cheaper, given that they would only be paying a salary and not full civil servants benefits. Some informants' (EM, DoH) accounts showed anxiety around the prospect of government absorbing huge numbers of LHWs. Two informants (DoH) showed suspicion towards those members of civil society who were pushing for the absorption of LHWs. These informants (DoH) suggested that NGOs may have hidden motives behind their positions, such as anxiety about limited donor funding. One informant suggested that this issue remained unresolved:

In terms of the model, the agreement has been *thus far*, that the government as far as possible do not *employ *community health workers but we fund NGO's to employ them. And to supervise them. That decision however is not favoured by civil society who think that that is a cop-out by government by asking NGOs to employ them; because once that NGO runs out of funding and then there's an issue. There's an issue of stability, there's an issue of supervision in the sense it follows the same process as '*casualisation' *of workers. And the feeling is that we, government, should employ them directly. So that's an issue that's unresolved in terms of our relationship with civil society (EM).

While some of the informants (Res, EM, DoH) suggested a polarisation with government on the one side wanting externalisation and civil society wanting absorption, the policy debate was more complex as the following quote suggests:

Some people who say no, it has to be state employment and to hell with NGOs... I think it's too difficult a battle because these NGOs will fight poisonously, so then let's go the other route and try to make the NGOs work properly. Turn them into proper primary health care promotion NGOs and not just vehicles for the externalisation of an employment relationship that the government is reluctant to take on itself (NGOD).

### Gender considerations in the policy redevelopment process

All of the informants agreed that LHWs were, in the majority, women and that the draft LHW policy (2009) pays considerable attention to ensuring that LHWs in general are not exploited. However, they also noted that the issue of gender is not linked to exploitation in the draft policy (2009) content. The absence of gender in the policy content was discussed in the interviews (Res, EM, DoH, NGOD). Some informants(EM, DoH) attributed the exclusion of gender in the draft policy (2009) content to competing priorities and also to the fact that policy issues tended only to be dealt with when the policy developers were confronted with these, such as when problems in the field were brought to their attention:

When we got confronted with an issue, we would then tend to explore the next issue and then we got into the legislative frameworks of that issue (DoH).

Only few of the informants (Res) referred to the growing literature on gender and LHWs. Some informants (DoH), particularly those responsible for policy development, were keenly aware of the findings of studies conducted locally by public health researchers whom were named in the interviews. Neither local studies which looked at gender specifically nor the agreements emanating from the 53 rd Commission on the Status of Women were mentioned by these informants (DoH). One informant (Res) suggested that policy makers may not know of or have read this material.

None of the informants, in their descriptions of the various influences and pressures on the policy process, suggested that there was any outspoken expectation to create and enforce a gender sensitive or gender redistributive policy. Furthermore, their descriptions of the policy actors did not suggest that there may have been individuals or groups who brought attention to the issue of gender in the policy process. This included a lack of influence of the office of the Gender Focal Point. Informants (DoH, EM, Res) claimed that this office did not participate in the development of this particular policy and that, in general, it was limited in its power and capacity to influence policy:

Every department *by law *needs to have a Gender Focal Point, which is an office. In some departments they report to the Minister and others the Director General. In our instance they report to the Director General, *their *role is to go through all policies and see if they are fair, gender neutral, if that's appropriate etcetera, but in *truth *the capacities of these officers are not strong. So you know, whether they have the *real capacity *to go through every policy to look at them, is an issue. But we have an office (EM).

Informants (EM) linked this incapacity for gender mainstreaming to a broader social context of patriarchy:

I don't think many of us know what it *means *to mainstream gender, you know we all talk about it as though we understand what it means but I'm not sure that we do. And that also means, you know, changing the attitude--changing what is essentially a patriarchal society (EM).

Some informants (Res, EM, NGOD), did however discuss these working conditions from a gendered perspective. These informants offered insights into a context of high unemployment which rendered women available to fill a gap in the health system without demanding huge payments. They (Res) also spoke about the hidden assumptions and expectations within policy and implementation that women should volunteer their time, linking this to an abdication of State responsibility and a transfer of this responsibility to poor and vulnerable women. Yet other informants (NGOD, Res) considered how the policy might enhance or limit female LHWs' access to training, education and career progression.

There was no indication from any of the informants that the issue of gendered exploitation or redressing gender inequity informed the debates around the policy problem. In trying to understand this one informant suggested:

Look the debates are not always formulated around the notion of the genderised nature of people. It's formulated around the exploitation. The gendered aspect is not always put into it. So in fact it's up to us people who are researchers to actually start to note that this is also related to the gendered notion of it (Res).

## Discussion

This paper has explored LHW policy redevelopment in South Africa and it has tried to understand the extent to which the issue of gender was taken up in this process. This stage of the policy process is referred to as the agenda setting phase during which issues get selected and prioritised to come onto the policy agenda [42,43]. Policy agendas are referred to as the "list of issues to which an organisation is giving serious attention at any one time with a view to taking some sort of action" [42].

The findings of the present study show that this policy redevelopment process was driven by a need to improve the working conditions of LHWs. This need was therefore on the top of the policy making agenda. Policy developers described how they came to understand the problem of LHW working conditions practically through field visits and empirically through research. However as the findings show the policy developers were not the only actors influencing the policy content and the redevelopment process. There were many actors and these actors all tried to ensure that their positions would be noted. Some of the actors had direct power to write the policy, while others exercised indirect power by trying to influence what was written. Not all actors were however happy with the level to which they were engaged or to the extent to which they felt heard. Overall the findings indicate that this process was robust and that the debates were active, even if actors did not always agree. Agenda setting has been described as being part of a process of health policy reform which involves many actors and interest groups [44,45]. As with other previous studies, the findings of our study have confirmed that these actors and interest groups exercise different degrees of power in influencing change or preventing change in the health policy reform process [44-47]. This power is particularly exercised in championing what issues will reach the policy agenda, in preventing other issues from being considered and in ignoring or not making a decision about certain issues [42,43,48,49]. Clearly the issue of reform of working conditions was championed, but our findings raise questions around the extent to which the issue of gender was championed.

The issue of gender did not enter the content or debate influencing the policy agenda. There was recognition by both policy makers and policy commentators that the working conditions arising from the implemented model of LHW governance and employment were problematic. The policy developers interviewed had attempted to address these working conditions through the development of the new policy content. However there was little recognition by policy developers interviewed that the problems with the working conditions were gender based and, in turn that this needed to be addressed as a gendered issue in the policy development process. Our data offers several possible explanations as to why this may have come about.

Even though national and international research points to the gendered issues around LHWs, policy developers interviewed were not aware of this body of work. It is not clear why this literature was not known but from the authors' own experiences the available research studies are not always easy to find. Most of these are available only as "grey literature" which was found by mining the internet and some of it was only obtainable directly from the authors themselves. A closer working relationship between policy makers and researchers may also have facilitated uptake of research into policy [50].

The findings also showed the lack of voice and power of LHWs as a collective in the contemporary policy redevelopment process. The literature points to the difficulty of organising LHWs as a collective group in South Africa [23], and during the course of the interviews there was no indication of the existence of an organised body LHW of representatives. The lack of participation in the policy process by ordinary people is not unique to the present policy process as it has been argued that public participation in agenda setting in developing countries has been limited [48]. There also did not appear to be a gender lobby engaging with the policy process and the role of the Gender Focal Point appeared to be limited. This suggests a possible failure of gender mainstreaming as a national government policy process to have an impact on the development of LHW policy. This reflects previous literature which describes the difficulties around the national gender machinery in ensuring and enabling gender sensitive policy [32].

Walt and Gilson [45] argue that policy is a process from development to implementation and that this process occurs within particular contexts with which it interacts. South Africa has a constitution which promotes gender equity and it has policies aimed at enabling gender sensitive policy making [31,33,51]. However, as one informant suggested, the South African context may still be that of patriarchy and within this context the process of enabling gender sensitive policy making is failing. Since this study did not explore the broader social context, this is hard to judge. None the less, some gender theorist argue that society is structured around a gender order which defines male and female responsibilities, and maintains the power of the privileged [52-54]. LHW policies and programmes cannot be said to be operating outside of this process. If recognised and acknowledged, policy can serve to either challenge or maintain this gender order.

It is important to note that the policy developers told that policy issues became important when they were confronted with them and when they were brought to their attention. This links to the literature on agenda setting in policy making which suggests that policy issues need a champion [49]. In the present policy process there were clearly champions around the issue of working conditions, but no-one linking the problems experienced by female LHWs to gender. This raises the question as to whether it is sufficient, in the context of a policy process such as this one, to challenge the structure of working conditions alone without recognising that this structure is particularly gendered. We would argue that this approach is limited as women in South Africa continue on many levels to be more vulnerable than men [4,55] and, in addition, women as care workers have been shown to be particularly vulnerable [20,23,56]. Furthermore, recent research on men has shown that engaging them in care-work can have important benefits [57]. For example, such engagement has been shown to challenge men's own conceptions of gender stereotypes and may encourage a change in their behaviour, thereby impacting ultimately on gender transformation [57]. Thus some consideration needs to be given to how this might be facilitated through policy, so as to enable transformation for women and for men both in general and in their roles as care-workers.

### Strengths and limitations of the study

A strength of this study is that it explores policy development beyond the written policy content and attempts to understand this process from the perspective of the actors engaged directly and indirectly with it. In doing so this study has drawn on informants across the policy development spectre and therefore is informed by a range of opinions and experiences which were not always in agreement with each other. This allows for a balanced reflection on the process. Another strength of this study is that we have described in the methods section several measures taken to establish the trustworthiness of the study.

Although it was difficult to get some of the informants to agree to be interviewed, in the end we felt that we had reached the key informants. The study would of course have been enhanced if we had reached even more informants, such as the politicians or persons from the Department of Labour. We compensated for this by doing in-depth interviews with those informants we had reached. A key limitation of the study is that it has not looked at the perspective of policy implementers (such as provincial programme managers or NGOs employing LHWs) nor has it explored the experiences and desires of LHWs themselves. However implementation and therefore the perspectives of implementers were not the focus of our research.

We found that individual in-depth interviews were the most appropriate data collection tool for this study. Given the profile of the informants, their geographical spread and the sensitivity of the topic, focus groups would have been difficult to perform. The authors also considered observing policy processes. However these processes are often diffuse, decision making and discussions often occur outside of formal meetings and government processes are not always open to the public.

As is the case of qualitative studies generalisations cannot be drawn in a statistical sense. However we hope that this study gives some insight into how the process around the design of a policy framework might be and why the gender perspective is not taken care of also in similar settings.

### Implications for future research

Future research could explore how to effectively include the voices of ordinary women, such as LHWs, in policy making for health and other areas. The response of LHW programme implementers (NGO staff and health services staff) is also important. The extent of research amongst such persons and what gaps there may be in this body of knowledge needs to be explored.

## Conclusions

LHW policy redevelopment was an active process, with many actors and strong debates focusing primarily on resolving issues of LHW working conditions. The issue of gender never reached the policy agenda of the present process. Primarily this seems to be because the issue of gender was not championed by anyone and policy developers subsequently did not take it on board. It was not sufficient to have national and departmental gender policies. Just as with any policy issue, the issue of gender needs champions.

## Abbreviations

AIDS: Acquired immune deficiency syndrome; DoH: National Department of Health officials; EM: Executive health managers; HIV: Human immunodeficiency virus; LHW: Lay health worker/s; LMICs: Low and middle income countries; NGO: Non-governmental organisation/s; NGOD: Nongovernmental organisation director; NPO: Non-profit organisation/s; Res: Researchers; WHO: World Health Organisation.

## Competing interests

The authors declare that they have no competing interests.

## Authors' contributions

KD conceived of the idea for the research. She was assisted in the design of the study and the research instruments by MC and KCR. KD conducted the interviews and led the analysis and writing under the guidance of MC and KCR. All authors read and approved the final manuscript.
